# Identification of features of electronic prescribing systems to support quality and safety in primary care using a modified Delphi process

**DOI:** 10.1186/1472-6947-10-21

**Published:** 2010-04-15

**Authors:** Michelle Sweidan, Margaret Williamson, James F Reeve, Ken Harvey, Jennifer A O'Neill, Peter Schattner, Teri Snowdon

**Affiliations:** 1 National Prescribing Service Ltd, Level 7, 418A Elizabeth St, Surry Hills NSW 2010, Australia; 2 School of Public Health, LaTrobe University, Bundoora VIC 3086, Australia; 3 Medical Software Industry Association Inc, PO Box 1293, Wahroonga NSW 2076, Australia; 4 Department of General Practice, School of Primary Health Care, Monash University, Notting Hill VIC 3168, Australia; 5 Royal Australian College of General Practitioners, 1 Palmerston Cres, South Melbourne VIC 3205, Australia

## Abstract

**Background:**

Electronic prescribing is increasingly being used in primary care and in hospitals. Studies on the effects of e-prescribing systems have found evidence for both benefit and harm. The aim of this study was to identify features of e-prescribing software systems that support patient safety and quality of care and that are useful to the clinician and the patient, with a focus on improving the quality use of medicines.

**Methods:**

Software features were identified by a literature review, key informants and an expert group. A modified Delphi process was used with a 12-member multidisciplinary expert group to reach consensus on the expected impact of the features in four domains: patient safety, quality of care, usefulness to the clinician and usefulness to the patient. The setting was electronic prescribing in general practice in Australia.

**Results:**

A list of 114 software features was developed. Most of the features relate to the recording and use of patient data, the medication selection process, prescribing decision support, monitoring drug therapy and clinical reports. The expert group rated 78 of the features (68%) as likely to have a high positive impact in at least one domain, 36 features (32%) as medium impact, and none as low or negative impact. Twenty seven features were rated as high positive impact across 3 or 4 domains including patient safety and quality of care. Ten features were considered "aspirational" because of a lack of agreed standards and/or suitable knowledge bases.

**Conclusions:**

This study defines features of e-prescribing software systems that are expected to support safety and quality, especially in relation to prescribing and use of medicines in general practice. The features could be used to develop software standards, and could be adapted if necessary for use in other settings and countries.

## Background

Medicine prescribing and health information record-keeping are changing from paper-based to computerised processes in healthcare systems all over the world. Electronic prescribing (e-prescribing) can be a stand alone process, but is usually part of an electronic health record system which may also link to pathology, radiology and patient administration systems. In the UK, Australia and some European countries the majority of prescribing in primary care is computerised, whereas uptake is less extensive in hospitals. In the United States, e-prescribing is widely used in some hospital-based organisations but is less common in primary care; this is likely to change with recent government incentives to encourage e-prescribing[1].

Most studies on the effects of e-prescribing systems have been carried out in the hospital setting, and have found evidence for both benefit and harm. E-prescribing produces legible prescriptions, can provide rapid access to information and decision support, and can reduce prescribing errors and adverse drug events [2-4]. It has also been associated with new types of errors and adverse patient outcomes [5-7]. The effects of e-prescribing on the quality of prescribing depend on a range of factors that include the healthcare setting, user training and behaviour, availability of appropriate hardware and technical support, the computer system used including the availability and quality of decision support, and integration of the system into work practices[8,9].

Numerous software applications have been developed for e-prescribing in the last 10 to 15 years, however guidance and standards for these systems has lagged behind development of the applications. The question of how these systems can maximise benefits and limit harm is receiving increasing attention. Some research has been done on the desirable functionality of these systems, [10-12] and on safety features[13,14]. Accreditation and certification programs such as the General Practice Systems of Choice program in the UK[15] and the Certification Commission for Health Information Technology in the United States[16] have been introduced. In Australia general practice prescribing systems have been used since the early 1990s, and in 2005 almost 90% of Australian general practitioners (GPs) were prescribing electronically[17]. The prescribing systems available have been developed without standards or accreditation and they have markedly different user interfaces and capabilities.

We undertook a study to identify features of prescribing systems used in general practice that support patient safety and quality of care and that are useful to the clinician and the patient, based on the consensus of an expert group. The focus was on the quality use of medicines (QUM i.e. judicious, effective and safe use of medicines): this is one of the four key objectives of the Australian National Medicines Policy, and the National Prescribing Service is the organisation that is responsible for promoting QUM. A list of the 114 features identified, together with their expected impact in four domains, is reported in this paper.

## Methods

A list of features was developed by the authors and an expert panel was convened to establish consensus on the expected impact of these features. Features included functional capabilities and other attributes of the prescribing system.

The study was conducted between November 2006 and December 2007 and was overseen by an 8-member study guidance group. The study protocol was approved by the Australian Department of Health and Ageing Ethics Committee.

### Identification of features

The features were identified by a literature review, key informant interviews and invited written submissions.

#### Literature review

Key papers and reports were identified by members of the project team who were familiar with work in this area. We searched Ovid MEDLINE(R) (Jan 1995 - Feb 2007) and used Google to search the internet to identify papers that covered features of e-prescribing systems that could significantly improve safety or quality, especially in relation to prescribing medicines and monitoring drug therapy. Scientific journals and grey literature were included. The papers and reports were assessed for relevance, and information about potential features was extracted.

#### Key informant interviews

We sought the opinions of Australian experts in general practice, public health, quality and safety, health informatics and pharmacy. A telephone interview guide was developed asking participants to identify current or potential features of electronic prescribing systems that could contribute to patient safety and quality patient care, or would be useful to the clinician or the patient. The interview guide was pilot tested with a user and non-user of e-prescribing systems, and was incorporated into a template to facilitate computer data entry during the interviews.

#### Written submissions

Written submissions were invited from members of the Medical Software Industry Association, the body representing general practice software vendors for most systems in use in Australia.

#### Development of features list

A list of candidate features was developed and was reviewed by five members of the project team (MW, MS, JR, KH, AS) in a series of round-table discussions. The inclusion of each feature was considered in terms of evidence or expectation that it would contribute to improved safety and quality or would be useful to clinicians or patients, and relevance to the general practice setting in Australia. For those features selected, some were merged, separated or the wording adapted for local relevance while maintaining the key concepts. The final wording of each feature was reviewed to ensure that it was clear, concise and accurate. The ability to implement each feature was considered - that is, whether it was possible to implement the feature given constraints such as lack of a suitable agreed standard or knowledge base. A rationale for each feature was developed based on the literature review and the clinical experience of the researchers, and the features were classified into convenient groups.

### Confirmation of features and rating of expected impact

A modified Delphi process was used with a 12-member multidisciplinary group of experts to gain consensus on the expected impact of the features. The Delphi process is used to predict effects where the available evidence is limited[18]. We used a modified process including face to face meetings because the project was complex and there were a large number of items to be scored. Similar techniques have been used in other studies to gain consensus on important features of information systems and quality of care indicators[19,20].

#### Expert panel recruitment

Participants were selected based on their experience in general practice, public health, quality and safety, health informatics, pharmacy or consumer health issues. Prior to the first meeting, each participant received information about the study and signed consent, confidentiality and declaration of interests forms. Participants were compensated for their time and travel expenses according to the organisation's remuneration policy.

#### Expert panel role

**Figure 1:**
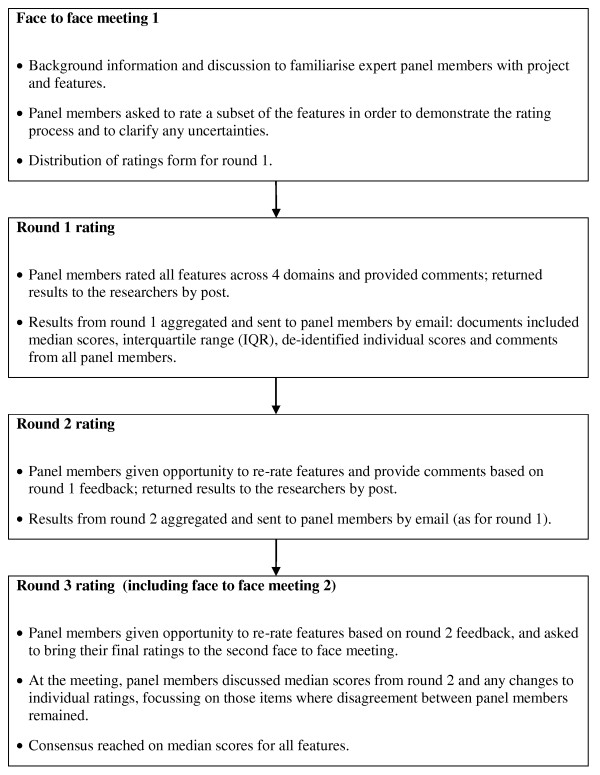
**Modified Delphi process**.

**Figure 2:**
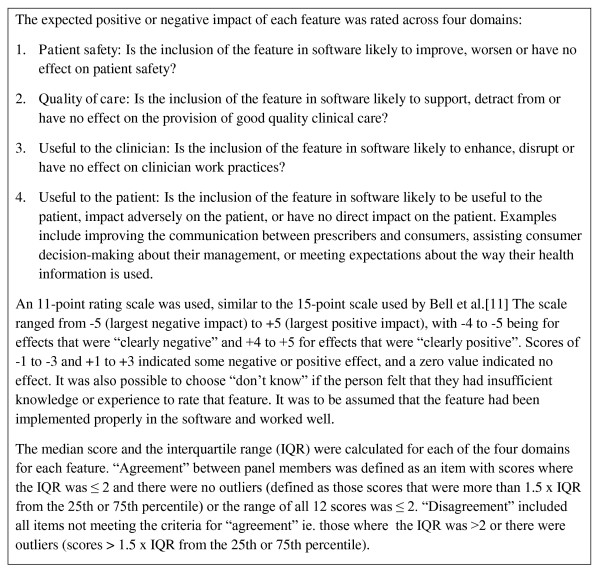
**Ratings system used by expert panel**.

**Figure 3:**
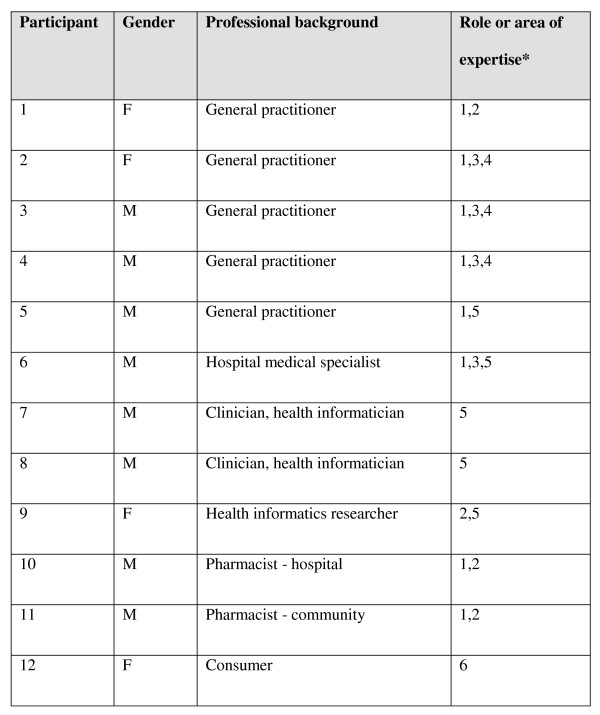
**Expert panel members**. *1 = clinical, 2 = academic, 3 = health policy, 4 = quality and safety, 5 = health informatics, 6 = consumer.

The panel members were asked to: (1) confirm that the proposed features were relevant, and add any other features that they considered important; (2) rate the expected impact of each feature (using a -5 to + 5 scale) across four domains, via a modified Delphi process (see Figures 1 and 2) and (3) define the cut off levels for categorising the features as being of high positive, medium positive or low/negative impact. The panel members were required to attend two meetings and complete three rounds of ratings over a two month period. The purpose of the first face to face meeting was to ensure that expert panel members understood the background to the project, the aims of the Delphi process and the scoring system. The purpose of the second meeting was to reach consensus on those items where agreement had not been reached in the third round of individual ratings.

## Results

### Identification of features

A list of 114 features was developed, selected from an initial list of over 200 candidate features; these were based on the review of the literature, key informant interviews and expert panel member recommendations.

#### Literature review

Six key literature sources[10-12,21-23] were used to inform the development of 102/114 (89%) of the features, with most of the features supported by more than one source. Of the six key sources, three of the studies or reports were related to prescribing in primary care or outpatient clinics, although they had different purposes. The first was a study to identify features which would reduce prescribing hazards,[10] the second identified features to improve patients' health outcomes and their ability to manage costs[11] and the third was intended to assist GPs in purchasing prescribing software systems[21]. The NHS *ePrescribing Functional Specification for NHS Trusts*[12] outlines e-prescribing specifications that are mostly applicable to the acute care setting although some are also relevant in primary care. The other two reports were published by international organisations and describe functionality or standards for electronic health record systems including prescribing. The Health Level 7 *Electronic Health Record Functional Model*[22] covers a "superset" of functions of an electronic health record system, intended to be adapted to different settings. The International Standards Organisation standard *Health informatics - Requirements for an electronic health record architecture *outlines clinical and technical requirements[23]. Eleven other references were also useful in the identification of relevant features[6,17,24-32].

#### Key informant interviews

Twenty-seven experts were contacted, 19 of these agreed to participate and 17 completed the interview. Their professional backgrounds were in general practice (6), health informatics or information technology (4), pharmacy (3), a medical specialty (2), nursing (1) or health policy (1), with many also having expertise in healthcare quality and safety. The key informants identified a broad range of features, with 62 of the features based on or supported by key informant comments.

#### Feedback from software vendors

General comments were received, most of them about the project methods and the need for industry standards in order to improve systems. These were not incorporated into the features list.

#### Preliminary features list

One hundred and seven features were selected for inclusion initially, with 7 added later by the expert panel (see below). The features were classified into eight groups: patient data (18 features), medication selection (24), decision support (22), patient information and education (6), monitoring, reports and recalls (17), interoperability and communication (13), security and administration (9) and transparency (5).

### Confirmation of features and rating of expected impact

#### Expert panel members

The 12-member expert panel was assembled from an initial pool of 23 potential participants, all based in Australia; their details are shown in Figure 3 and the Acknowledgments. Those who were not selected were either not available (7) or declined to participate (4). All 12 members completed the entire ratings process. The face to face meetings were attended by at least 75% of the panel members, with input via teleconference or email for those members unable to attend in person.

#### Expert panel ratings and recommendations

The expert panel recommended that all 107 features on the preliminary list be retained, and suggested the addition of seven new features, making a total of 114 features. Panel members rated the expected impact of each feature across four domains: patient safety, quality of care, usefulness to the clinician and usefulness to the patient (see Figure 2). At the end of round 1 there was agreement among panel members (defined in Figure 2) for 55% of the items rated. Feedback on results from round 1 (median scores, interquartile range (IQR), de-identified individual scores and comments) was provided to panel members prior to round 2 and members were asked to confirm their ratings or to re-rate their original scores in light of the feedback. In round 2 there was agreement for 78% of the items rated. In round 3, consensus was reached on the median scores for all 114 features in each of the four domains.

The panel recommended cut off points for classifying the expected level of impact of each feature as follows: high positive impact = at least 1 of the 4 median scores is 4-5; medium positive impact = at least 1 of the 4 median scores is 2-3.5 (and none are ≥ 4); low positive or negative impact = all four median scores are <2.

### Final features and their expected impact

The complete features list including the expected impact in each of the four domains is shown in Additional file 1. Most of the features relate to the recording and use of patient data, the medication selection process, prescribing decision support, monitoring drug therapy or clinical reporting. Other areas are resources for patients, interoperability, security and transparency. Seventy eight features (68%) were classified as likely to have a high positive impact in at least one domain, 36 features (32%) as medium impact, and none as low or negative impact. Twenty seven features (24%) were rated as high impact across three or four domains including safety and quality; these are highlighted in Additional file 1 and are described in the following two paragraphs. Ten features were considered "aspirational", that is they could not be implemented at the time of the study, either because of a lack of agreed standards or lack of nationally accepted knowledge bases being available in a suitable format.

The 27 features of e-prescribing systems that were considered likely to have high positive impact across multiple domains (including quality and safety) include the following: The system should allow the user to record patient clinical details, medication information, allergies and pregnancy and breastfeeding status; this information should be recorded in a format that can be used for decision support, and should be displayed to the user. When there are changes to or discontinuation of medications, the system should allow the date, prescriber and reason/s for change or discontinuation to be recorded. Apart from direct entry of data into the patient record, it should be possible to import clinical data from external sources and also to export it to external sources, allowing details of the record to be shared and updated. As part of the medication selection process, the system should offer the user information on recommended therapeutic options for the condition being treated, and provide access to evidence-based drug information at the time of prescribing. The system should display medication lists in a way that makes it easy for the user to differentiate between similarly named products, to reduce the risk of product selection errors. The system should also provide information on drug strength, form and dosage, and display the generic name when a product is selected by brand name.

All decision support should be underpinned by high quality, up to date knowledge bases and guidelines, and alerts should be prioritised by clinical importance. Warnings should be provided when prescribing a drug if: that drug (or another drug in the same class) is already on the current medications list; the patient has a recorded contraindication to the drug, including allergy, pregnancy or breastfeeding; the drug is renally cleared and the patient has renal impairment; the dosage regimen may be harmful; or where the regulator has issued a safety warning for a product. Finally, the system should enable the user to produce a suitably formatted current medication list for the patient, and should use standard clinical terminologies and messaging protocols.

## Discussion

One hundred and fourteen features of general practice prescribing systems that support quality and safety were defined, with a focus on promoting better use of medicines. All features were rated by a multidisciplinary expert panel as being likely to have medium to high positive impact on safety, quality of care and/or usefulness to the clinician or the patient. Ten "aspirational" features were identified; they require significant system changes before they can be implemented, for example availability and use of national clinical terminologies and unique patient identifiers. Many of these are essential "building blocks" for e-health, and are necessary for sharing patient data, for example a shared medication list.

The strengths of our study include the comprehensive approach and the focus on safety and quality, particularly in relation to medicines use. The features were developed and rated drawing on multiple sources, including the international literature and experts with different backgrounds. An expert panel reached consensus on the expected impact of the features not only on patient safety and quality of care, but also on whether they were likely to be useful to clinicians and to patients.

The scope of our features list has some limitations. The features were developed for the Australian general practice setting, although we believe most of the features are relevant to e-prescribing in other settings. Non-clinical functions of software were largely excluded, for example billing and practice management. The features are high level statements rather than detailed specifications; if they are used as the basis for specifications, factors such as usability and the quality of knowledge bases used for decision support also need to be considered.

Our results endorse the importance of many of the features that others have identified in different settings as being important for safety and quality in e-prescribing. We took a similar approach to Avery et al.[10] in the UK and Bell et al.[11] in the United States, with a common goal in all three studies being to support patient safety. Our study focussed on the quality use of medicines in general practice in Australia; however, our list of 114 features includes related aspects of prescribing systems that we believe are important, for example security and back-up of clinical data, transparency and support for privacy legislation. Avery et al.[10] produced 55 clinical statements related to medicines management errors and safety considerations for general practice computer systems in the UK, and their importance was rated. Bell et al.[11] developed 60 recommendations for e-prescribing systems' ability to meet the goals of improving patient safety and health outcomes, helping patients manage costs, maintaining patient privacy and promoting clinicians' acceptance of electronic prescribing. The recommendations were rated by level of impact on each of these four dimensions.

While the rating of our features cannot be compared directly with those of Avery et al.[10] and Bell al[11] (as different scales were used), there was considerable overlap in the areas rated as important. In Avery et al's study the key themes around which consensus was reached on importance included computerised alerts; the need to minimise spurious alerts; making it difficult to override critically important alerts; having audit trails of such overrides; effective computer-user interface; and the need to be able to run safety reports[10]. For the 60 recommendations made by Bell et al, 52 were rated in the clearly positive range on the 'patient safety and health outcomes' dimension, including all 19 features related to patient identification, medication selection and alerts and warnings for prescribing[11].

The features list produced in this study could be used by policymakers and professional bodies to develop certification criteria or standards for prescribing software systems, and as guidance for software vendors. It could also be used by clinicians to assist them in assessing the suitability of an e-prescribing system when selecting or changing systems.

Several lines of follow on work suggest themselves. Detailed specifications or criteria can be developed for incorporating (or testing) the features in e-prescribing systems. (Following this study we assessed seven e-prescribing systems in Australia to find out whether the features had been implemented in these systems; the results will be reported in the near future). It would be valuable to observe the actual impact of each feature when implemented. We would emphasise the importance of monitoring and evaluating the effects of e-prescribing systems when they are introduced or changed, being especially vigilant for unintended effects. Finally, new functionality will become possible as technologies and systems evolve; any list of recommended software features requires ongoing review.

## Conclusions

This study defines the features of e-prescribing systems that are expected to support safety and quality, with a focus on prescribing and use of medicines in general practice. The definition of such features is important for the development of software standards, with the aim being to maximise the benefits and minimise potential harms associated with e-prescribing. Software standards and certification processes are required to ensure that the features are incorporated into e-prescribing systems. The features list could be adapted for use in other settings and countries.

## Competing interests

JO represented the Medical Software Industry Association on the study guidance group; the MSIA covers more than 80 commercial companies with products and services in health IT.

## Authors' contributions

MW, MS and JR developed the idea for the study. All authors contributed to the study design. MS, MW, JR and KH reviewed the literature and administered the Delphi process with the expert panel. All authors reviewed the results. MS drafted the manuscript. All authors revised the manuscript critically and approved the final manuscript.

## Pre-publication history

The pre-publication history for this paper can be accessed here:

http://www.biomedcentral.com/1472-6947/10/21/prepub

## Supplementary Material

Additional file 1 **Features list including expected impact in 4 domains**. Shows the 114 features and the expert panel ratings for each feature. Expected impact: H = high positive impact, M = medium positive impact, L = low or negative impact * Features marked with an asterisk were rated by the expert group as high positive impact across 3 or 4 domains including safety and quality. ** Features marked with two asterisks were classified as "aspirational". † Indicates an Australian agency or entity; there may be equivalent agencies or entities in other countries. Click here for file
